# Review of the injectate dispersion pattern during anterior quadratus lumborum block

**DOI:** 10.1097/MD.0000000000032038

**Published:** 2022-12-02

**Authors:** Jin Wu, Yifan Qin, Huiyu She, Rui Ma

**Affiliations:** a Department of Anesthesiology, Affiliated Hospital of Jiangsu University, Zhenjiang City, Jiangsu Province, China; b Department of Human Anatomy, Medical School, Jiangsu University, Zhenjiang City, Jiangsu Province, China.

**Keywords:** anterior quadratus lumborum block, dispersion, mechanism, review

## Abstract

Since its introduction in 2013, the anterior quadratus lumborum (QL) block (QLB) has been widely used for analgesia in abdominal, pelvic, and hip surgeries, based on the assumption that it has analgesic effects on both incisional and visceral pain. However, the mechanism of the anterior QLB remains unclear, and the results of relevant studies are contradictory. This review aimed to summarize the dispersion patterns of injectates in anterior QLB. We conducted literature searches using PubMed, Cochrane, and Embase databases. A total of 10 cadaveric or radiological studies meeting the inclusion and exclusion criteria were summarized. The dye or contrast agent spread to the thoracic paravertebral space in only 5 of the studies reviewed. Variability in the selection of injection sites relative to the anterior layer of the thoracolumbar fascia during anterior QLB implementation may explain the dispersion difference. The correct injection site of an anterior QLB is anterior to the QL muscle and between the QL muscle and the anterior layer of the thoracolumbar fascia. Further studies are needed to verify the injectate dispersion pattern during anterior QLB.

## 1. Introduction

In 2007, Blanco first defined the anterolateral quadratus lumborum (QL) block (QLB) during the annual European Society of Regional Anesthesia Congress.^[1]^ In 2013, Børglum et al modified this technique by injecting a local anesthetic into the fascial interspace between the QL and psoas major (PM) muscles.^[2]^ This technique has been termed transmuscular QLB, QLB type 3, or anterior QLB in the years following its use. In 2021, the “anterior QLB” was recommended as the term for blocks in which the injection is situated in the plane between the QL and PM muscles during the first American Society of Regional Anesthesia and Pain Medicine-European Society of Regional Anesthesia and Pain Therapy International Nomenclature Delphi study.^[3]^ In the past decade, various clinical studies have shown that anterior QLB can produce good analgesic effects in abdominal, pelvic, and hip surgeries.^[4–8]^ The widespread use of the anterior QLB in clinics is based on the assumption that it has an analgesic effect on incisional and visceral pain simultaneously. This notion is based on observations in cadaveric studies that the dye spreads to the thoracic sympathetic trunk and the lower thoracic segmental nerves in the thoracic paravertebral space, as well as the subcostal, iliohypogastric, and ilioinguinal nerves, which run anterior to the QL muscle.^[9,10]^ However, the exact mechanism of action of the anterior QLB is unclear and the results of relevant studies are contradictory. In this review, we summarize anatomic and radiologic studies to clarify the dispersion pattern of the injectate in anterior QLB procedures.

## 2. Methods

The initial literature search was conducted in December 2021. We used the following databases: PubMed, Embase, and Cochrane Library. The second literature search was conducted in April 2022 to ensure that no new articles were missed. During the primary literature search, the following search terms were used individually or in combination: “anterior quadratus lumborum block,” “transmuscular quadratus lumborum block,” “quadratus lumborum block type 3,” “cadaver,” “cadaveric,” “anatomy,” “anatomic,” “radiology” and “radiologic.” Secondary searches were conducted using the bibliographies of articles identified in the primary search. The exclusion criteria were as follows: animal studies; studies not published in English as the first language; and reviews or systematic reviews.

## 3. Results

Ten eligible studies that satisfied all inclusion and exclusion criteria were retrieved. These included 3 case reports,^[2,11,12]^ 5 descriptive studies,^[9,10,13–15]^ and 2 randomized controlled trials^[16,17]^ (Table 1). The subjects in 3 studies were patients,^[2,12,17]^ whereas those in the remaining 7 studies were cadavers. When patients were enrolled as research subjects, the dispersion range of contrast was determined using imaging methods such as computed tomography or magnetic resonance imaging. When cadavers were used as research subjects, the dispersion range of the dye or contrast was determined by anatomical dissection,^[9,10,14–16]^ imaging,^[11]^ or both.^[13]^ Contrast or dye was injected through needles in all studies except 1 in which contrast was injected through a pre-embedded catheter.^[12]^

**Table 1:** Summary of the anterior QL block technique and injectate dispersion in the retrieved literature.

Study	Subjects	Needle tip position	Results
Børglum J, et al 2013^[2]^	Patient (2 blocks for MRI)	In the fascial interspace between the QL and PM muscle at L4 level after the ventral proper fascia of the QL muscle was penetrated	Contrast spread cranially along the QL and PM muscle, reaching the arcuate ligaments and beyond.
Carline L, et al 2016^[16]^	Cadavers (4 specimens for dissection)	Between the QL and PM muscle beyond the ATLF, anterior to the L3 or L4 TP	Dye spread consistently to the L1 and L3 nerve roots and within the PM and QL muscles.
Dam M, et al 2017^[9]^	Cadavers (14 specimens for dissection)	In the plane between the QL and PM muscle at L4 level (10 specimens) or at L2 level (4 specimens) after the investing fascia of the QL muscle was penetrated	Dye spread into the thoracic paravertebral and intercostal spaces through the pathway posterior to the medial and lateral arcuate ligaments. The subcostal, iliohypogastric and ilioinguinal nerves were dyed in all cases, whereas the genitofemoral and lateral femoral cutaneous nerves were dyed to varying degrees. The lumbar plexus, femoral nerve, or lumbar sympathetic trunk were not dyed.
Kumar A, et al 2017^[11]^	Cadaver (1 specimen for continuous fluoroscopic imaging)	In the space between the QL and PM muscle at L3 level	Contrast did not spread into the paravertebral space.
Elsharkawy H, et al 2017^[10]^	Cadavers (6 specimens for dissection)	Anterior to the QL muscle, and between the QL muscle and the ATLF at the L1-2 level	Dye spread to the iliohypogastric and ilioinguinal nerves, subcostal nerves, T9 to T12 and L1 nerve roots, with variable staining of the higher thoracic nerve, up to T5.
Adhikary SD, et al 2017^[13]^	Cadavers (10 specimens for dissection and 8 for CT)	In the fascial plane between the QL and PM muscle close to the L3 TP after the ventral fascia of the QL muscle was penetrated	Injectate spread over the posterior abdominal wall to the PM muscle and the upper branches of the lumbar plexus. Spread to the thoracic paravertebral space was not radiographically evident.
Sondekoppam RV, et al 2018^[14]^	Cadavers (10 specimens for dissection)	Ventral to the QL muscle at L3 level after the anterior layer of the QL fascia was penetrated	Dye spread consistently to ilioinguinal, iliohypogastric, and subcostal nerves with variable staining of the T9–T11 nerves laterally in the transversus abdominis plane and transversalis fascia. Thoracic paravertebral spread was identified only in 2 specimens.
Elsharkawy H, et al 2019^[15]^	Cadavers (12 specimens for dissection)	Between the QL and PM muscle close to the L5 TP	Dye spread consistently to the majority of the branches of the lumbar plexus, including the femoral, lateral femoral cutaneous, ilioinguinal, and iliohypogastric nerves. Ten specimens exhibited thoracic paravertebral dye spread to the T10 level. No specimens exhibited L5 or sacral nerve root staining or caudal spread below the L5.
Diwan S, et al 2019^[12]^	Patients (5 blocks for CT)	Catheter tip in the ATLF at the level of the kidney	Contrast spread to the thoracic paravertebral and epidural space with high anterior QLB. A catheter tip position between the mid and lower pole of the kidney resulted in a more caudal and lateral spread.
Balocco AL, et al 2021^[17]^	Patients (20 blocks for CT)	In the plane between the QL and PM muscle proximal to the L2 TP	Contrast spread consistently in the anterior aspect of the QL muscle with occasional spread to the lumbar and thoracic paravertebral areas.

ATLF = anterior layer of the thoracolumbar fascia, CT = computed tomography, MRI = magnetic resonance imaging, PM = psoas major, QL = quadratus lumborum, TP = transverse process.

The needle tip was most often positioned at the L3 or L4 level,^[2,9,11,13,14,16]^ followed by the L2 level^[9,17]^ or L1-2 level,^[10]^ and at least at the L5 level.^[15]^ Only 1 study placed the ultrasound probe in the parasagittal oblique plane and inserted the needle in-plane from the caudal to cranial direction through the QL muscle.^[10]^ In the remaining 9 studies, the probe was placed in a transverse orientation on the abdominal flank cranial to the iliac crest or in a transverse oblique paramedian orientation lateral to the lumbar spinous process. The needle was inserted in a posterolateral to anteromedial or posteromedial to anterolateral direction, penetrating the QL muscle.^[2,9,11–17]^ However, the exact location of the tip of the needle remains unclear. The needle tip has been described as embedded in the fascial plane between the QL and PM muscles,^[2,9,13]^ between the QL and PM muscles,^[11,14–17]^ between the QL muscle and the anterior layer of the thoracolumbar fascia (ATLF),^[10]^ or in the ATLF.^[12]^ The majority of studies did not specify whether the ATLF penetrated.^[2,9,11,13–15,17]^ Four studies reported that the needle tip approximated the transverse process.^[13,15–17]^

The dye or contrast spread to the thoracic paravertebral space consistently or in the majority of subjects in 4 studies.^[2,9,10,15]^ The dye or contrast only spread occasionally or did not spread to the thoracic paravertebral space in 5 studies.^[11,13,14,16,17]^ One study reported that contrast spread to the thoracic paravertebral space was dependent on the level at which the anterior QLB was performed.^[12]^ Spread of the dye to the lumbar nerve roots was observed in 1 study,^[16]^ whereas spread of the dye or contrast to the branches of the lumbar plexus, such as the iliohypogastric and ilioinguinal nerves, was observed in 5.^[9,10,13–15]^ No study has reported staining of the sacral nerve root.

## 4. Discussion

The thoracolumbar fascia is a blend of the aponeurotic and fascial planes that form the retinaculum around the paraspinal muscles of the lower back and sacral regions. The cranial and caudal sides are connected to the endothoracic and iliac fasciae, respectively, which can potentially ensure the diffusion of local anesthetics. In the 3-layer model of thoracolumbar fascia, the anterior layer is defined as the fascial band that passes anterior to the QL muscle and ends by turning posteriorly to pass between the QL and PM muscles. ATLF has been described as an extension of the transverse fascia. Cross-sectionally, the QL muscle is typically anteromedially overlapped by the PM muscle, and the ATLF covering the QL muscle ventrally separates it from the PM muscle.^[18]^ The ATLF splits into 2 layers at the level of the arcuate ligament of the diaphragm. One layer is continuous with the endothoracic fascia and the second is the inferior diaphragmatic fascia. Therefore, theoretically, when the injection site is between the QL muscle and ATLF during the anterior QLB procedure, the injectate can spread into the lower thoracic paravertebral space via the arcuate ligaments. Clinical research has demonstrated that the higher the injection point, the higher the maximum cephalad dermatome level reached.^[19]^ In a modified subcostal anterior QLB immediately below the lateral arcuate ligament, the injectate spreads cranially via the posterior pathway of the lateral arcuate ligament on the sonogram.^[20]^ In a cadaveric study, where the dye was injected specifically between the QL muscle and ATLF, it was observed that the dye could spread to the thoracic paravertebral space.^[10]^ Therefore, this reported mechanism of anterior QLB injectate dispersion is anatomically plausible and has been demonstrated in clinical practice.

However, half of the studies on the dispersion of injectates during anterior QLB did not support this mechanism.^[11,13,14,16,17]^ The reasons for the contradictory results may include disregarding the existence of the ATLF between the QL and PM muscles or a misinterpretation of the correct injection site in the application of the anterior QLB. For example, in 1 study, the injection site was outside the ATLF.^[16]^ Other studies failed to determine whether ATLF had penetrated.^[11,13,14,17]^ The ATLF is 0.10 mm thick, and sometimes cannot be clearly appreciated under ultrasound imaging. In 5 studies,^[12–16]^ the needle entry path passed from the posterolateral to the anteromedial side of the QL muscle until it reached the space between the QL and the PM muscles. It easily penetrates the ATLF during needle insertion. No analgesic effects were observed when the injection site was fat in the preperitoneal space between the QL and PM muscles.^[21]^ When the injection site approximates the PM muscle, it results in a block of the adjoining lumbar nerve roots or lumbar plexus.^[13]^

This study has 2 major limitations. First, the variability of the study design, research subjects, and injectate disposition in the retrieved literature did not allow for a meaningful statistical analysis of the distribution patterns of injectates during anterior QLB. This reduced the effectiveness and authority of the review. Second, we only reviewed the mechanisms of the anterior QLB based on the commonly held belief that a more medial and deeper anterior QLB would result in a more extensive spread toward the paravertebral space and sympathetic chain than the lateral and posterior QL blocks.

In summary, the dye or contrast agent spread to the thoracic paravertebral space in only 5 (50%) of the 10 studies reviewed. Variability in the injection sites relative to ATLF in the implementation of the anterior QLB might have been the reason for the difference in dispersion. The optimal injection site to facilitate the anterior QLB is anterior to the QL muscle and between the QL muscle and the ATLF. Further studies are needed to verify the injectate dispersion pattern during anterior QLB.

## Author contributions

**Conceptualization:** Jin Wu, Rui Ma.

**Data curation:** Yifan Qin, Huiyu She.

**Funding acquisition:** Jin Wu.

**Methodology:** Yifan Qin, Huiyu She.

**Writing – review & editing:** Jin Wu, Rui Ma.
